# Electrospark Deposition and Ultrasonic Peening Treatment on AlSi10Mg Powder Bed Fusion–Laser Beam Parts: Microstructure and Properties

**DOI:** 10.3390/ma19102041

**Published:** 2026-05-13

**Authors:** Paola Leo, Gilda Renna, Andrea Amleto De Luca, Riccardo Nobile, Caterina Casavola, Vincenzo Moramarco, Simone Carone, Michele Angelo Attolico

**Affiliations:** 1 Department of Engineering for Innovation, University of Salento, Via per Monteroni, 73100 Lecce, Italy; 2 Dipartimento di Meccanica, Matematica e Management, Via Orabona 4, 70125 Bari, Italy

**Keywords:** Electrospark Deposition, ultrasonic peening treatment, AlSi10Mg, additive manufacturing, microstructure, properties, defects, corrosion

## Abstract

Additive manufacturing (AM) has revolutionized industrial production. However, the repair of AM components remains a critical challenge due to their unique microstructural features. While repair approaches for conventionally manufactured alloys are well established, their direct transferability to AM parts remains largely unexplored due to the unique thermal history and anisotropic microstructure of additive components. This study investigates a novel repair and improvement strategy for Powder Bed Fusion–Laser Beam/Metal (PBF-LB/M)-fabricated AlSi10Mg components, combining Electrospark Deposition (ESD) for dimensional restoration with subsequent Ultrasonic Peening Treatment (UPT) for surface enhancement. Microstructure, porosity, surface roughness, hardness profiles, residual stresses, and corrosion behaviour were systematically characterized using SEM, optical microscopy, profilometry, Vickers microhardness testing, XRD, and electrochemical polarization tests. The results show that the ESD process is capable of producing coatings with excellent interfacial adhesion to the substrate, with an initial porosity of 3.6 ± 0.5%. The subsequent UPT induces a significant densification effect on the deposited material, reducing porosity by approximately 50% and increasing surface hardness by up to 48% in the upper region of the coating. Furthermore, XRD analysis reveals that UPT completely reverses the residual stress state from tensile (typical of the ESD process) to compressive in all measured directions, thereby improving the overall structural integrity. Ultimately, the combined ESD + UPT alters the electrochemical response of AlSi10Mg deposits, resulting in a nobler corrosion potential, albeit with a slightly higher corrosion current density.

## 1. Introduction

The role of repairs of metallic components has become essential in mitigating degradation that can compromise component performance and shorten service life in high-demand applications. Repair processes for the refurbishment of materials derived from traditional manufacturing have been extensively studied and optimized over the years, including both manual and automated processes for heavy industrial equipment, highlighting their potential to extend lifecycle and reduce waste [1,2,3,4].

In recent years, these aspects have been further investigated through the application of additive manufacturing techniques such as Cold Spray and Directed Energy Deposition (DED) for the repair of damaged components produced from both traditionally and additively manufactured alloys (e.g., AA7050 [5], A356-T6 [6], Ti6Al4V [7], X2CrNiMo17-13-2 [8]). While these repair methods have proven effective for large-area refurbishment of such components, the coating and dimensional restoration of small-scale damage in additively manufactured parts remains insufficiently explored. Moreover, the repair of localized defects or the restoration of dimensional accuracy can become highly costly and time-consuming when components must be disassembled and transported off-site for conventional or advanced treatments. In such cases, in situ repair represents a more cost-effective, time-efficient, and practical alternative, as it minimizes downtime and enables on-site refurbishment.

This need for effective repair solutions is particularly relevant for components produced by Powder Bed Fusion–Laser Beam/Metal (PBF-LB/M), one of the most mature and widely adopted laser powder bed fusion technologies for metals. PBF-LB/M enables the layer-by-layer fabrication of complex components from a broad range of metallic materials, including iron-, copper-, aluminum-, and titanium-based alloys, as well as cobalt- and nickel-based superalloys [9]. This advanced technique integrates laser-based powder fusion with rapid prototyping principles. It enables the production of parts and components with highly complex geometries, such as lightweight lattice structures, integrated internal channels, and intricate conductive architectures [9,10]. Therefore, the PBF-LB/M process offers advantages in both overall manufacturing and the production of complex parts compared with traditional metal processing technologies [11].

Due to the unique microstructural features of PBF-LB/M-produced components, which arise from the extremely high cooling rates (typically 10^3^–10^8^ K/s [12,13,14]), the selection of appropriate repair technologies is crucial. The deposited material must exhibit microstructural characteristics similar to those of the damaged substrate to ensure compatibility and minimize adverse effects on the base material during the repair process.

As reported in a previous study [15], Electrospark Deposition (ESD) is an innovative technology that has attracted considerable interest in the field of component repair, thanks to its numerous advantages, including low environmental impact, the ability to perform deposition at atmospheric pressure, and the ease of handling and portability of the equipment.

Widely used for the deposition and repair of surface cracks and localized defects in metals, ceramics, and metal matrix composites [16,17,18,19], ESD is an environmentally sustainable (“green”) surface engineering technology. The process relies on pulsed spark discharges between a rotating electrode and a conductive substrate, which induce localized melting at the electrode tip. Through electrode rotation, the molten droplets are subsequently transferred onto the substrate surface, resulting in a surface-alloyed deposition layer characterized by excellent metallurgical bonding and minimal alteration of the base material microstructure [20]. Specifically, the technique involves plasma generation during spark discharge, rapid localized melting and solidification, material transfer from the electrode to the substrate, and interfacial diffusion processes, all of which govern the formation and properties of the deposited layer. Due to the extremely high cooling rates achieved (typically on the order of 10^5^–10^6^ K/s [21]), ESD produces coatings with an ultrafine microstructure and features closely resembling those observed in PBF-LB/M processes [16,17,22]. In addition, it has been found that surface roughness after ESD repair can be improved by applying Ultrasonic Peening Treatment (UPT). Surface quality plays a critical role in the service life, reliability, and durability of additively manufactured parts, particularly under cyclic loading or in aggressive environments, where surface defects act as stress concentrators and crack initiation sites [23].

UPT is an advanced cold-working technique derived from shot peening and is considered one of the most effective methods for the surface treatment of metallic materials. It introduces significant compressive residual stresses into the near-surface layer while simultaneously reducing surface roughness, increasing fatigue resistance, and closing or minimizing porosity [24,25,26]. The system typically comprises an ultrasonic power generator, a transducer with a horn, and a specially designed pin or rod attached to the horn tip. During the process, high-frequency (typically 20 kHz), low-amplitude ultrasonic vibrations are transmitted through the pin or rod directly to the workpiece surface, inducing severe plastic deformation in a localized manner [25].

Studies on UPT, including applications on AA7050 [27] and AlSi10Mg [28], have demonstrated its effectiveness for both conventionally and additively manufactured materials. Consistently, improvements in fatigue resistance, corrosion resistance, microhardness, and overall surface integrity have been reported. These benefits arise from the continuous high-frequency ultrasonic impacts of the tool on the workpiece surface, which generate substantial compressive plastic deformation, accompanied by surface grain refinement and material strengthening [24].

Despite its proven effectiveness in enhancing surface integrity, UPT presents several practical challenges when applied manually, including the need for precise control of treatment angle, pressure, and duration to achieve uniform coverage, as well as operator fatigue induced by high-frequency vibrations and ultrasonic noise.

In recent years, increasing attention has been devoted to the processability of Al alloys via PBF-LB/M. Among the broad range of Al alloys, the hypoeutectic AlSi10Mg alloy has attracted particularly strong interest owing to the critical role of the Al–Si eutectic system [29,30]. Its near-eutectic composition provides excellent weldability, high melt fluidity, and low solidification shrinkage, making it especially well suited to the extreme cooling rates characteristic of PBF-LB/M. These conditions promote the formation of an ultrafine microstructure characterized by a continuous network of eutectic Si surrounding the α-Al matrix, resulting in superior mechanical properties, such as higher hardness and strength, compared with conventionally cast counterparts [31]. This fine eutectic structure is further enhanced by Mg, which contributes to additional strengthening through solid-solution effects and precipitation hardening during ageing treatments. Moreover, Al-Si alloys such as AlSi10Mg exhibit excellent electrical and thermal conductivity [32].

The literature has comprehensively investigated AlSi10Mg across all key stages: from powder characteristics and process parameter optimization [22,33,34] to microstructural tailoring and mechanical property enhancement [35,36], up to topology-optimized component design and validation of functional performance in realistic operating conditions [37,38,39]. For these reasons, AlSi10Mg was selected as the reference material for the present study, which focuses on surface coating and localized repair via ESD, further enhanced by UPT.

Although the literature extensively documents the refurbishment of traditionally manufactured components using both conventional and innovative techniques, to the best of the authors’ knowledge, there is very limited evidence regarding the application of ESD for the repair and dimensional restoration of PBF-LB/M-produced components. Evidence is even more limited for the use of UPT on additively manufactured parts. The present work aims to explore the potential of combining ESD and UPT techniques for the refurbishment of PBF-LB/M AlSi10Mg components. To this end, the effects on microstructure, mechanical properties, corrosion behaviour, and residual stress distribution will be systematically analyzed. The authors believe that the outcomes of this study may provide valuable insights and contribute to advancing knowledge in the field of post-processing and repair of additively manufactured metallic parts.

## 2. Materials and Methods

**Figure 1:**
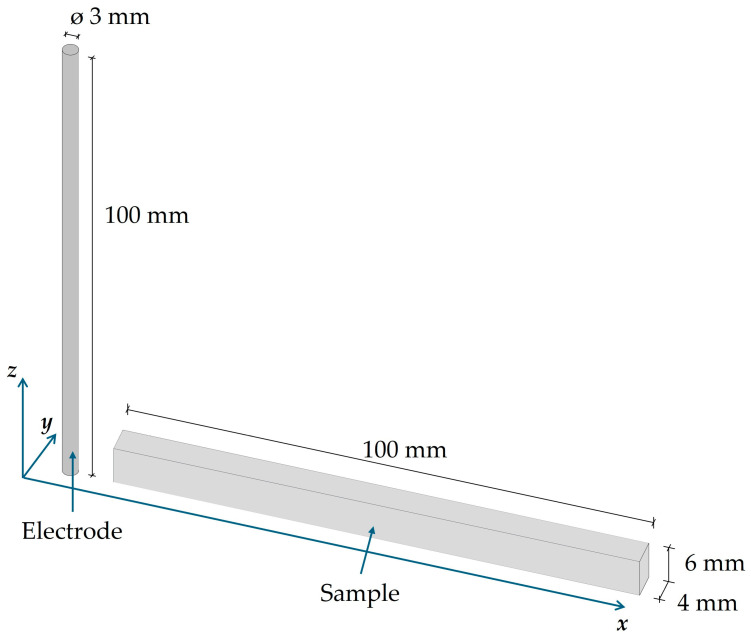
AlSi10Mg sample and electrode geometries produced by PBF-LB/M.

Gas-atomized AlSi10Mg powder was employed for the fabrication of specimens and electrodes by PBF-LB/M using a Renishaw AM 400 machine (Renishaw plc, Wotton-under-Edge, UK). A meander scanning strategy with 45° rotation between consecutive layers was adopted. The chemical composition of the powder is provided in Table 1, and the PBF-LB/M process parameters are detailed in Table 2. The AlSi10Mg samples were fabricated using a build platform preheated to 200 °C to minimize thermal gradients and reduce the accumulation of residual stresses during the PBF-LB/M process. Following fabrication, the samples underwent a post-process heat treatment at 300 °C for 2 h, followed by air cooling. Sample and electrode geometries are illustrated in Figure 1.

**Figure 2:**
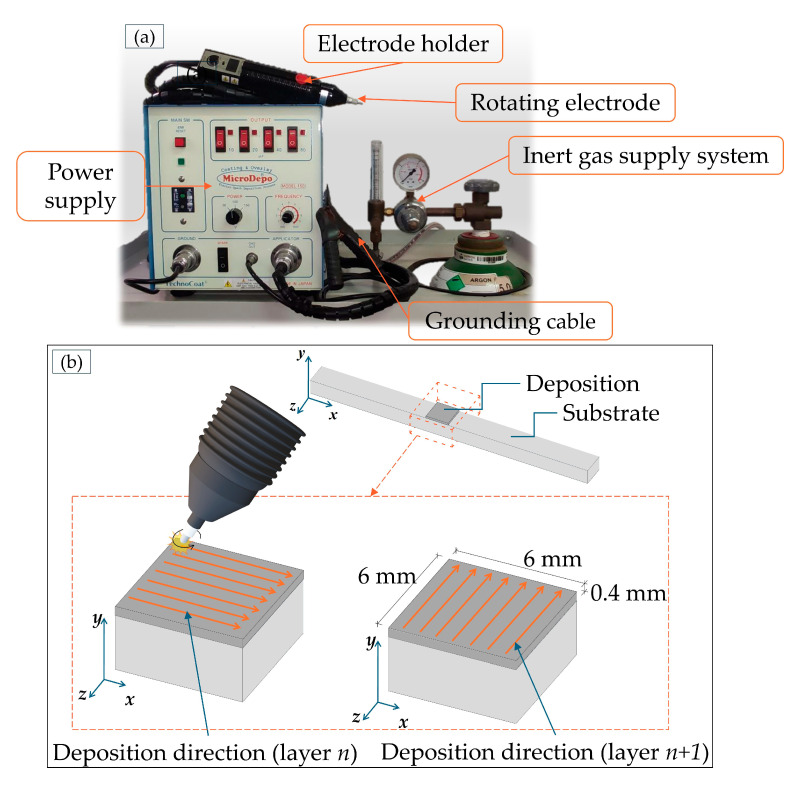
(**a**) Components of ESD instruments; (**b**) schematization of deposition strategy applied by ESD on AlSi10Mg substrates.

For Electrospark Deposition, a 6 × 6 mm^2^ area on the AlSi10Mg As-Built and heat-treated substrate was treated using a Technocoat MicroDepo Model 150 machine (TechnoCoat Co., Ltd., Fujieda, Japan), as shown in Figure 2a. The ESD process parameters voltage (*V*), capacitance (*C)*, and frequency (*f*), reported in Table 3, were selected following a parameter optimization procedure. The spark pulse energy (*E_s_*) and power (*P*), also listed in Table 3, were calculated as follows:


(1) $$E_{S}=\;\frac{1}{2}\;C\cdot V^{2}$$

(2) $$P=\;E_{S}\cdot f$$

The ESD process was carried out manually using the electrode holder shown in Figure 2a at room temperature under a continuous argon flow of 15 L/min, thereby minimizing contamination of the deposited material by interstitial elements such as oxygen and nitrogen. Once the substrate was fixed in position, deposition was performed using orthogonal scanning passes between the *n*^th^ and (*n* + 1)^th^ layers (Figure 2b) to achieve a more uniform coating and to avoid significant surface waviness. A final coating thickness of 0.4 mm was obtained through successive overlapping depositions in the build (Y) direction. The aim was to achieve an adequate deposition rate while ensuring good adhesion both to the substrate and between successive layers.

**Figure 3:**
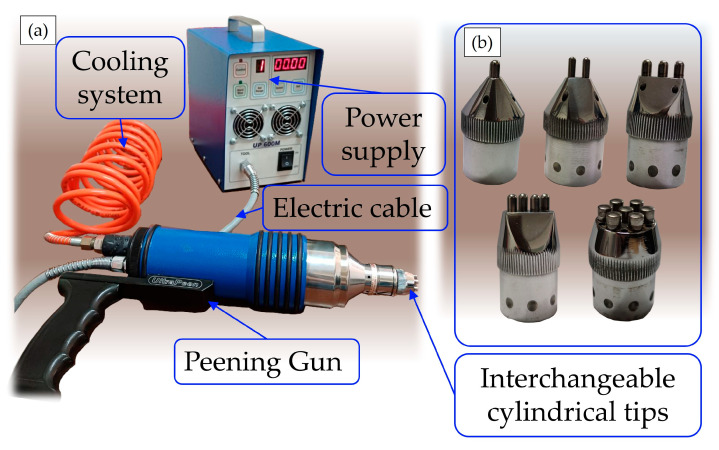
Components for Ultrasonic Peening Treatment: (**a**) UPT machine; (**b**) different designs with interchangeable UPT cylindrical tips.

Ultrasonic Peening Treatment (UPT) was applied to the ESD coatings using a UP 600M Syntes system (SINTEC Inc., Markham, Canada), as shown in Figure 3a. The equipment allows the selection of four ultrasonic energy levels: 1 (minimum), 2 and 3 (intermediate), and 4 (maximum). The treatment duration can also be adjusted according to specific requirements. Depending on the surface condition, different pin configurations can be employed using interchangeable working heads (Figure 3b). In the present study, a three-pin-in-line tip was mounted on the peening gun. The detailed UPT process parameters are summarized in Table 4.

To compare their microstructures and properties, samples were examined in as-built and heat-treated (300 °C for 2 h) (AB samples) condition, after Electrospark Deposition (ESD samples), and after subsequent Ultrasonic Peening Treatment (ESD + UPT samples). Microstructural characterization was performed using a NIKON EPIPHOT 200 optical microscope (Nikon, Tokyo, Japan) and a ZEISS EVO scanning electron microscope (Zeiss, Oberkochen, Germany) equipped with a Bruker energy-dispersive X-ray spectrometer (EDX) (Bruker Nano GmbH, Berlin, Germany). Metallographic cross-sections were prepared along the *xy* plane. After polishing, the samples were chemically etched using Keller’s reagent (95 mL H_2_O, 2.5 mL HNO_3_, 1.5 mL HCl, 1 mL HF) to reveal the microstructure. Optical micrographs were acquired and analyzed using Nikon NIS-Elements software (6.10.02 version). Porosity content and void distribution within the coating were quantified from binarized images using ImageJ software (1.54g version).

Roughness analysis was performed using a fringe projection system based on non-contact optical measurements. The setup consisted of a 2 MP camera (1624 × 1234 pixels), a fringe projector, and a computer for data processing. The fringe projection technique reconstructs the 3D surface topography of an object by analyzing the deformation of a projected fringe pattern captured by the camera sensor [40]. A sequence of sinusoidal fringe patterns was projected onto the specimen surface, and the captured images were processed to extract the phase map *ϕ*(i,j) at each pixel, which was then converted into 3D (*x*, *y*, *z*) coordinates of the surface [41,42]. For each sample, an area of 3 × 3 mm^2^ was analyzed. A Gaussian filter was applied to remove the waviness component from the profile, thereby isolating the roughness contribution. This filtering procedure was calibrated against a reference sample previously characterized with a MarSurf PS10 contact roughness profilometer (Mahr GmbH, Göttingen, Germany). Surface measurements were repeated five times per sample to ensure repeatability.

For residual stress measurement, the guidelines provided by the technical standard UNI EN 15305 (2008) [43] were followed. Residual stresses were measured at the midpoint, in the *xy* plane, of the considered areas for the AB substrate and for those subjected to ESD and UPT surface treatments, along three directions defined by angles of 0°, 45°, and 90° relative to the *x*-axis of an arbitrarily established reference system in the measurement plane. This approach was chosen due to the absence of predefined preferred orientations for residual stress analysis. The measurements were conducted using the Xstress 3000 G3R diffractometer (STRESSTECH) (Stresstech Oy, Vaajakoski, Finland) in a modified χ configuration, employing Cr Kα radiation and a Miller index (*hkl*) of 311. The device was equipped with a 2 mm circular collimator, and each measurement had a 30 s exposure time at a tube voltage of 30 kV and a tube current of 6.5 mA. Measurements were taken at tilt angles of 0°, ±24.1°, ±35.3°, and ±45°, with stress values calculated using the sin^2^ψ method. X-ray diffraction from the Miller (311) family of crystallographic planes was analyzed using the Pearson VII profile interpolation function, with a diffraction angle (2θ) of 139.3°. Elastic constants for the analysis were set to ½ S2 = −4.887 × 10^−6^ MPa^−1^ and S1 = 1.905 × 10^−5^ MPa^−1^.

Vickers hardness was measured for ESD and ESD + UPT samples using an Affri Wiky 200JS digital instrument (Affri, Varese, Italy), employing 0.1 kg for a holding time equal to 15 s (HV_0.1/15_) [44]. In particular, hardness measurements were performed on *xy* cross-sections along the *x* direction, starting from the substrate and extending through the entire coating up to the top surface. At each depth, a minimum of five measurements were taken along the *y* direction, and the average value was then calculated.

The corrosion resistance of the AB, ESD and ESD + UPT samples was evaluated using a Gamry 1010E potentiostat (Gamry Instruments, Warminster, PA, USA). Electrochemical measurements were carried out in an aqueous 3.5 wt.% NaCl solution at room temperature under naturally aerated, near-neutral conditions. A conventional three-electrode electrochemical cell was employed, consisting of an expanded graphite mesh as the counter electrode, a Ag/AgCl electrode as the reference electrode, and the samples as the working electrode. All the potentials were referenced to the Ag/AgCl electrode. To ensure accurate measurements, the samples were electrically insulated using Teflon, while the electrical connection was established via a copper wire soldered to the specimen, covering the cut edges and back surfaces and leaving only a 1.0 cm^2^ surface area exposed to the electrolyte. Since one of the main objectives of this study was to evaluate the surface quality of the treated samples, no grinding or polishing was performed on the exposed surface prior to the potentiodynamic tests. The steady-state potential was determined after immersing the samples in the electrolyte at open circuit potential (OCP) for 15 min. Subsequently, potentiodynamic polarization tests were conducted by scanning the potential from −1.5 V to +1.5 V vs. Ag/AgCl at a scan rate of 2 mV s^−1^.

## 3. Results and Discussion

### 3.1. Microstructure Characterization Before and After UPT

**Figure 4:**
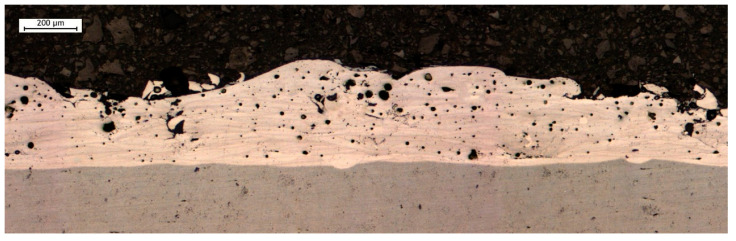
Cross-section (XY plane) of the coating deposited by the ESD technique on the AlSi10Mg substrate.

Figure 4 shows the cross-section of the deposition obtained using the ESD technique. The coating–substrate interface appears continuous and is characterized by a high degree of integration, suggesting excellent metallurgical bonding. This excellent adhesion is particularly promising for dimensional restoration of PBF-LB/M components, where structural integrity is paramount. Nevertheless, localized porosity is observed within the deposited layers. Specifically, Figure 4 highlights two main pore sizes and morphologies, which will be discussed in detail in the following paragraph.

**Figure 5:**
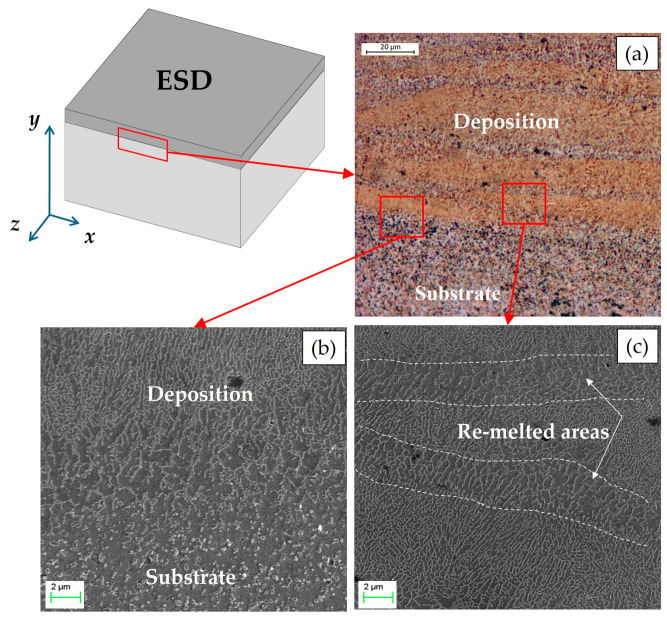
Microstructure of the deposition obtained by the ESD technique: (**a**) optical micrograph at 500× magnification; (**b**,**c**) SEM micrographs at 10,000× magnification.

The optical micrographs (Figure 5a) and SEM image (Figure 5b), taken after chemical etching with Keller’s reagent, highlight excellent adhesion, continuity, and the absence of delamination between the deposited layers and at the layer/substrate interface.

The microstructure of the ESD-deposited layer consists of columnar cells predominantly aligned parallel to the build-up direction, as shown in Figure 5c. This morphology closely resembles the typical microstructure observed in Al-Si alloys produced by PBF-LB/M [22,45].

Another similarity to the PBF-LB/M process is the partial remelting of previously deposited and solidified layers [45,46]. Each single deposition layer is clearly visible in Figure 5a, as it appears with a darker contrast. Moreover, each layer is formed on a remelted area, which appears lighter. These areas are further highlighted in Figure 5c, where a coarser microstructure can be observed as a direct consequence of remelting. In contrast, the adjacent regions exhibit a much finer microstructure, which is a consequence of the extremely high cooling rates (10^5^–10^6^ K/s) inherent to the ESD process [20,21,47]. Specifically, the highest cooling rate is expected for the first deposited layer, which is in direct contact with the colder substrate, leading to the formation of the finest grain structure in this zone.

**Figure 6:**
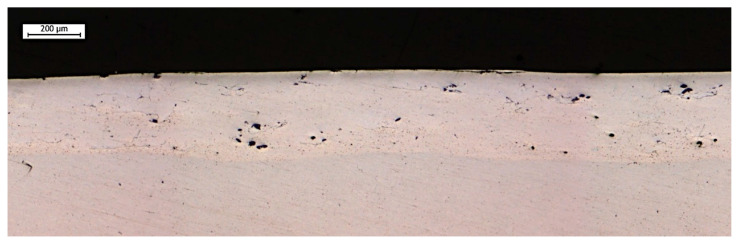
Cross-section (Plane XY) of ESD + UPT coating on AlSi10Mg substrate.

Figure 6 shows the cross-sectional view of the ESD coating after Ultrasonic Peening Treatment. A clear reduction in porosity and a significant flattening of the surface can be observed. The decrease in surface waviness is attributed to the ultrasonic impact of the pins on the working head, which effectively reduces surface asperities [25].

**Figure 7:**
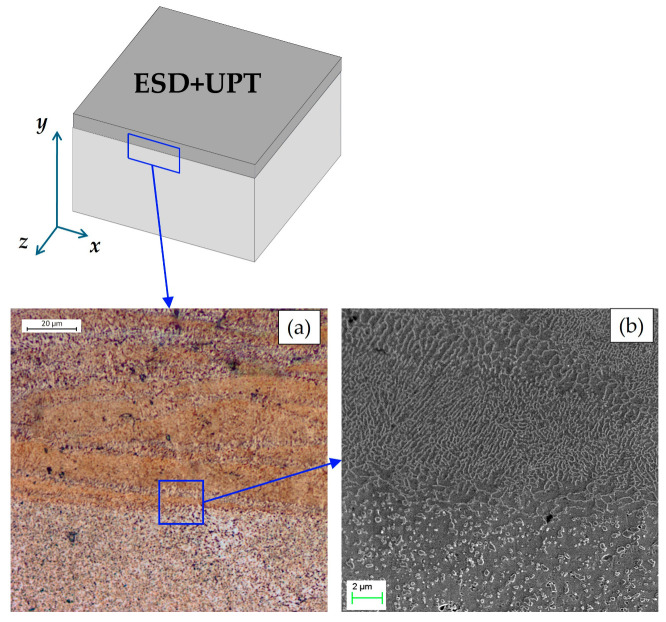
Microstructure of the ESD coating after UPT at the substrate–deposition interface: (**a**) optical micrograph at 500× magnification; (**b**) SEM micrographs at 10,000× magnification.

The optical micrograph in Figure 7a and the SEM image in Figure 7b show the interface region between the substrate and the ESD coating after UPT. The characteristics of this interfacial zone are essentially unchanged compared to those observed prior to the ultrasonic treatment.

**Figure 8:**
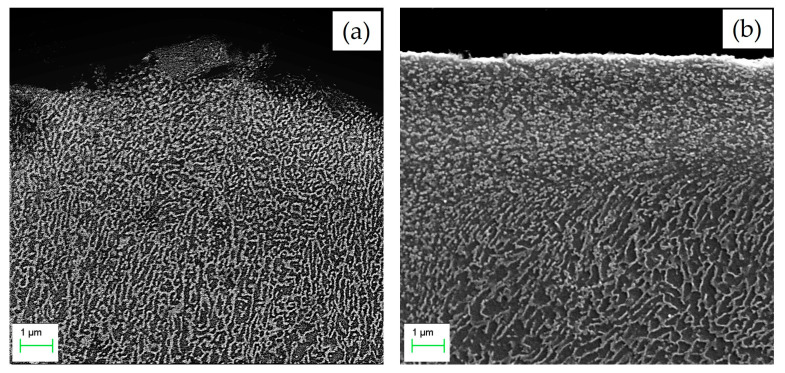
SEM micrographs at 20,000× magnification of the upper edge of the deposition: (**a**) as-deposited ESD coating; (**b**) ESD coating after UPT.

In contrast, significant differences are observed near the top of the coating cross-section before and after UPT (Figure 8). The SEM image in Figure 8b clearly shows a flattening of the coating’s upper edge together with a significant evolution of the microstructure. As confirmed by [25,26,27], ultrasonic surface treatments can induce both particle fragmentation and grain refinement effects in the near-surface region of the treated material.

### 3.2. UPT Effects on Properties Evolution

Ultrasonic Peening Treatment applied to the ESD coatings resulted in a clear evolution of both surface morphology and subsurface properties. First, a reduction in the coating height (thickness) was observed after UPT: the mean height decreased from 390 ± 40 µm (as-deposited) to 300 ± 25 µm post-UPT. This thickness reduction reflects the significant plastic strain induced in the deposited material via ESD. At the same time, the imposed plastic strain promotes strain hardening in the treated region and, as reported in similar studies [26], leads to the subdivision of the original grains into smaller sub-grains (grain refinement). Consequently, a significant increase in hardness is achieved.

**Figure 9:**
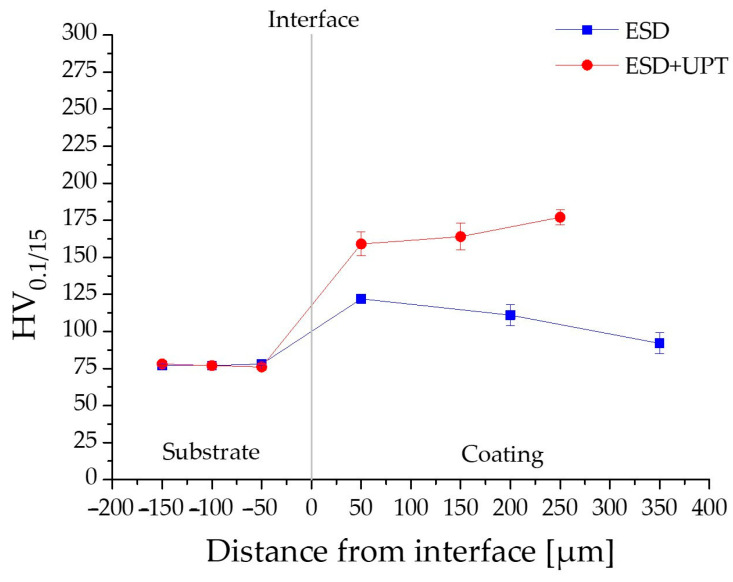
Comparison of microhardness profile (HV_0.1/15_) before and after UPT.

Figure 9 compares the microhardness evolution across the cross-sections of the samples in both the as-deposited (ESD sample) and post-treated (ESD + UPT sample) conditions.

Regarding the ESD sample in the as-deposited state, a progressive decrease in microhardness is observed from the substrate/coating interface toward the surface layers of the coating. In particular, microhardness measurements performed near the substrate/coating interface (at a distance of 50 μm within the coating, as shown in Figure 9) indicate an average value of 122 ± 3 HV. This peak hardness corresponds to the first deposited layers, which experience the highest cooling rates due to rapid heat dissipation through the cold, bulk substrate. After this, within the coating, microhardness progressively decreases with increasing deposition height, reaching 111 ± 7 HV in the middle layers and 92 ± 7 HV at the upper layers. The observed microhardness reduction in the middle and upper layers of the ESD coating, compared to the layers close to the substrate interface, can be attributed to the progressive heat accumulation during sequential deposition of several layers. As a result, the first layers benefit from a steep thermal gradient and rapid heat dissipation through the cold substrate, whereas subsequent layers are deposited onto an increasingly warmer surface. This heat accumulation effect in the previously deposited layers increases the average temperature of the growing coating and, consequently, reduces the cooling rate ($\dot{T}$). In solidification theory, the cooling rate is defined by the product G⋅R (where G is the thermal gradient and R is the solidification interface rate), which governs the characteristic scale (or size) of the solidification microstructure, while the ratio G/R governs the resulting solidification morphology. In general, a higher cooling rate promotes finer structures, whereas a lower cooling rate promotes microstructural coarsening. Accordingly, variations in G and R along the coating height lead to differences in microstructural scale throughout the deposit. Therefore, in the upper regions, the reduced cooling rate promotes microstructure scale growth (microstructural coarsening), resulting in lower hardness values (Figure 9) [15,48,49].

In contrast, the substrate exhibits a stable hardness of 78 ± 1 HV, fully consistent with literature values for PBF-LB/M-fabricated AlSi10Mg subjected to heat treatment at 300 °C for 2 h [22,50,51].

According to the literature, ultrasonic surface treatments result in a substantial increase in the hardness of the treated material. Previous investigations consistently report that the degree of this hardening effect decreases as the distance from the treated surface increases [26,28,52,53]. This trend is clearly observed in the ESD + UPT samples of the present study, as illustrated by the microhardness profiles in Figure 9. Specifically, following Ultrasonic Peening Treatment, a hardness gradient is still present across the coating thickness; however, it is reversed compared to that in the as-deposited ESD condition. While the hardness increase extends throughout the entire coating thickness, the most pronounced enhancement is observed in the upper layers, near the treated surface. In this area (Figure 8b), the highest level of work hardening is achieved due to the intense plastic strain induced by UPT. Moving towards the substrate, the hardness values progressively decrease. Notably, the hardness measured in the substrate region immediately next to the interface (at a distance of −50 µm) remained essentially unchanged following the UPT process. This indicates that the mechanical energy of the peening treatment was effectively dissipated within the coating volume, achieving significant densification without affecting the bulk properties of the underlying PBF-LB/M-fabricated component.

Moreover, the application of UPT also resulted in a significant improvement in surface roughness. This pronounced smoothing effect can be attributed to the repeated high-frequency impacts of the peening pins, which promote homogenization of the surface [25]. This result agrees with a study by Chen et al. [52], in which it was reported that UPT is the most effective treatment in reducing roughness compared to other peening techniques. In the present work, UPT produced a marked decrease in surface roughness of the ESD coating. The average Ra values obtained using the fringe projection technique are reported in Table 5.

**Figure 10:**
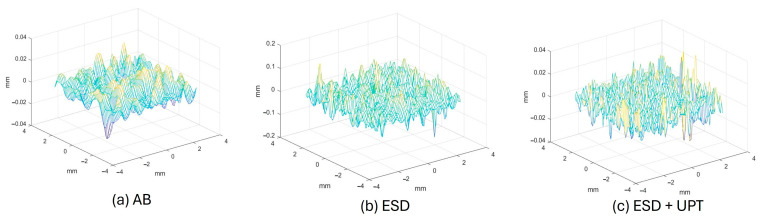
Comparison of 3D surface topography maps illustrating the morphological evolution: (**a**) as-built (AB) PBF-LB/M AlSi10Mg substrate, (**b**) as-deposited Electrospark Deposition (ESD) coating, and (**c**) surface finish after combined ESD and Ultrasonic Peening Treatment (ESD + UPT).

Following UPT, the surface roughness of the coating approached that of the as-built PBF-LB/M substrate, whose average Ra was equal to 6.66 ± 0.36 µm. The 3D surface maps in Figure 10 visually confirm the transition from the inherent texture of the as-built substrate (Figure 10a) to the highly irregular, crater-like morphology of the as-deposited ESD coating (Figure 10b), which exhibits the highest vertical peak-to-valley distance. The subsequently UPT-treated surface (Figure 10c) is clearly flattened, resulting in a significantly more uniform topography and a dramatic decrease in surface roughness.

Another significant effect of Ultrasonic Peening Treatment is the substantial reduction in porosity [25,28]. This aspect is very significant concerning ESD coating. Indeed, the amount of porosity in terms of area percentage is quite high after ESD and equal to 3.6 ± 0.5%. The smaller circular pores are derived from entrapped gas that cannot escape from the melt pool due to the high solidification rate [18]. Moreover, larger pores are typically induced by ineffective filling (with the melted droplets) of the waviness in the previously deposited ESD layer, leading to the occurrence of empty areas.

**Figure 11:**
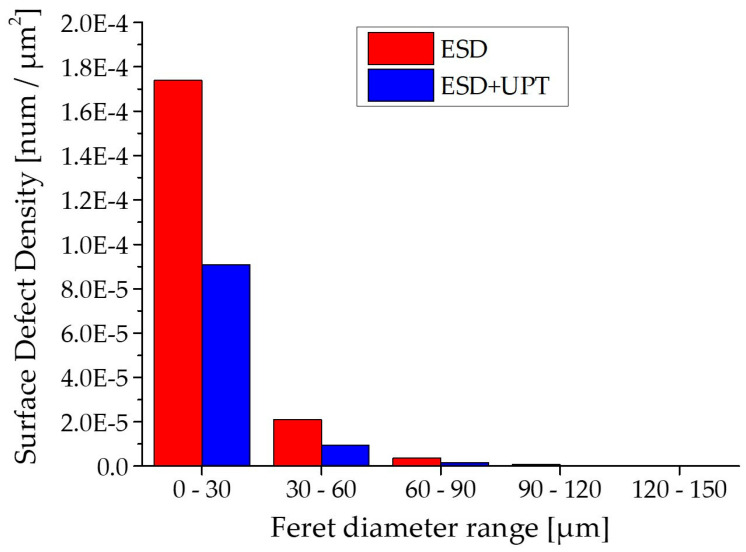
Void surface density in various Feret diameter ranges in ESD and ESD + UPT samples.

During the UPT application, the high-frequency impacts close the voids, causing a decrease in porosity. The average area porosity percentage of ESD + UPT was evaluated at 1.8 ± 0.5%, which is half of the porosity level in the ESD coating. This result was due to a reduction in the pore number in all diameter ranges investigated, as shown in Figure 11. Moreover, a change in the shape of the pores has been observed, according to the study of Gou et al. [54], who proposed that UPT, when it does not succeed in closing the coarsest existing pores, can still modify their shape by squeezing. Table 6 reports the distribution of void percentages within the coating according to circularity ranges. The void distribution with respect to the circularity range changes after UPT, leading to a decrease in the number of more circular voids (circularity range 0.801–1).

**Figure 12:**
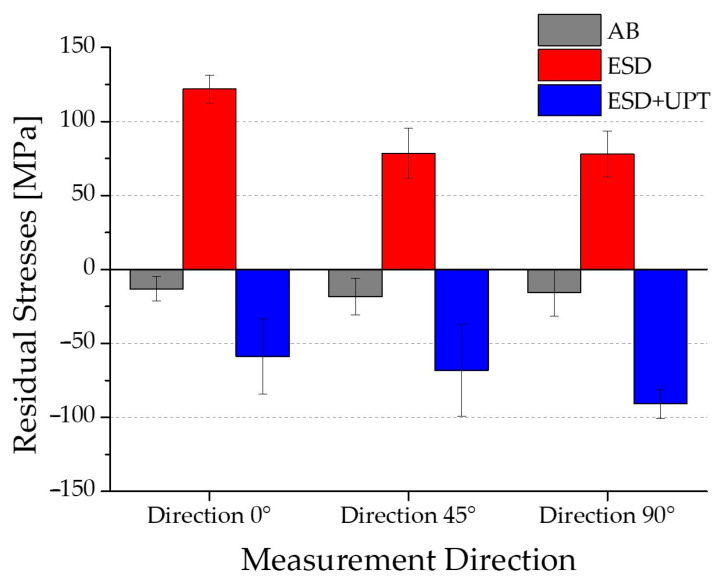
Trend of residual stresses in ESD and ESD + UPT samples.

The extremely high cooling rate characteristic of the Electrospark Deposition (ESD) process typically generates tensile thermal stresses within the coating [15,55]. Our experimental measurements confirm this phenomenon and demonstrate the effectiveness of the subsequent treatment: as shown in Figure 12, the residual stress state of the ESD coating was significantly modified by the Ultrasonic Peening Treatment (UPT). The compaction action during UPT was sufficiently intense to completely reverse the tensile stresses originally present in the as-deposited ESD coating. This reversal was consistent across all three measurement directions (0°, 45°, and 90°), where the stress state shifted from approximately +100 MPa (tensile) to values as low as −90 MPa (compressive). These findings are consistent with literature reports indicating that induced compressive residual stresses are beneficial for mechanical performance, especially in terms of fatigue strength [25,56], as they hinder crack initiation and propagation.

### 3.3. Corrosion Behaviour

**Figure 13:**
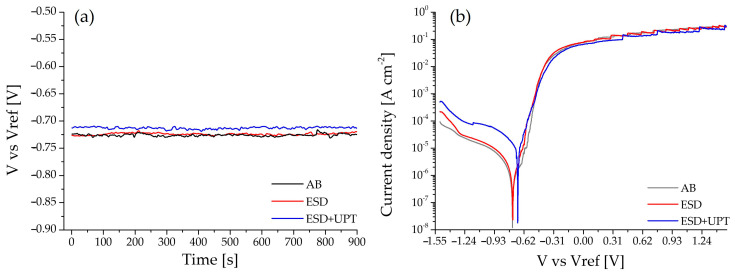
(**a**) OCP and (**b**) potentiodynamic curve comparison among AB, ESD and ESD + UPT samples.

Corrosion performance was evaluated by electrochemical polarization tests on samples in the as-built and heat-treated (300 °C for 2 h) state (AB sample), after Electrospark Deposition (ESD sample) and after electro-discharge deposition and Ultrasonic Peening Treatment (ESD + UPT sample). The open circuit potential (OCP) evolution and the potentiodynamic polarization curves are reported in Figure 13a,b, while the main electrochemical parameters, namely, corrosion potential (E_corr_) and corrosion current density (j_corr_), are summarized in Table 7.

As observed in Figure 13a, all samples (AB, ESD, and ESD + UPT) exhibited a highly stable OCP from the earliest stages of immersion, indicating that the surfaces rapidly reached an electrochemical equilibrium in the 3.5 wt.% NaCl solution. The AB and as-deposited ESD samples display similar OCP values, characterized by a more active (less noble) potential compared to the UPT-treated condition. In particular, the ESD + UPT sample shows a slightly more noble potential (approximately −0.71 V) than the AB and ESD samples (around −0.73 V). This upward shift, although small, suggests a surface condition that is more resistant to the onset of corrosion.

Consistent with the OCP results, the corrosion potential (E_corr_) of the ESD + UPT sample shifted toward more positive values (−0.69 V) compared to the AB sample (−0.74 V) (Figure 13b and Table 7), indicating a shift in the electrochemical equilibrium toward nobler conditions. A slight increase in j_corr_ was observed for the ESD + UPT sample (from ~2.718 10^−6^ to ~1.6863 10^−5^ A/cm^2^, approximately one order of magnitude higher than the AB sample), which could indicate a higher corrosion rate from a purely kinetic standpoint. Considering these results, further investigations are warranted and will be addressed in future work. In particular, pre- and post-corrosion analyses by SEM/EDS will be essential to better elucidate the degradation mechanisms of the ESD + UPT coatings, as well as the role of the increasing dislocation density induced by severe plastic deformation.

The observed improvement in E_corr_ can be associated with the reduced surface roughness (Table 5), deposit densification (Table 5), microstructural evolution (Figure 8b) and compressive residual stress state [57] (Figure 10) induced by Ultrasonic Peening Treatment.

The transition from tensile to compressive residual stresses modifies the near-surface atomic configuration by increasing lattice compaction, which can affect the interfacial electrochemical activity. In contrast, tensile stresses, characteristic of the as-deposited ESD condition, promote lattice expansion and facilitate localized electrochemical activity [57]. Concurrently, UPT imposes severe plastic deformation, leading to fragmentation and refinement of the eutectic Si phases.

As previously established, the ESD-deposited AlSi10Mg microstructure, analogous to that of PBF-LB/M-processed AlSi10Mg, is intrinsically heterogeneous, consisting of a fine cellular dendritic microstructure within the layers interspersed with layer-layer interfaces (traces) characterized by equiaxed morphology and coarser Si-rich phases [16,47]. These interfacial regions are well known to act as preferential sites for galvanic interactions due to the electrochemical contrast between the α-Al matrix and Si-enriched phases, whereas the finer cellular structure within the layers provides comparatively more homogeneous electrochemical behaviour [58,59]. The refinement and redistribution of Si-rich phases induced by UPT disrupt the continuity of these galvanic micro-cells, thereby promoting a more homogeneous electrochemical response [60]. As a result, the combined ESD + UPT leads to a modified surface state characterized by reduced heterogeneity compared to the ESD condition.

Finally, it is worth noting that the electrochemical behaviour of the AB substrate and that of the as-deposited ESD coating remain comparable. This apparent convergence arises from the competing influence of heat treatment performed at 300 °C for 2h on the PBF-LB/M sample. While this heat treatment decreases residual stresses, it simultaneously drives significant microstructural evolution. Specifically, although melt pool boundaries remain visible and the initial cellular morphology is only partially preserved, such thermal exposure promotes the decomposition of the Si network (Si-network breakdown), with Si particles becoming spheroidized and coarsening through Ostwald ripening [61,62], along with an increase in the spacing between neighbouring Si clusters. As a result, the average size of Si-rich regions increases at the expense of their number density [63]. Therefore, this microstructural evolution becomes the dominant factor, ultimately impairing the intrinsic corrosion resistance of the AB sample. The significant decline in corrosion resistance for PBF-LB/M AlSi10Mg samples treated at 300 °C compared to untreated as-built samples has been extensively documented. Xinhui Gu et al. [64] reported that the corrosion current density of untreated PBF-LB/M AlSi10Mg samples is approximately one-fifteenth of that of samples heat-treated at 300 °C, demonstrating that the supersaturated Al matrix with a continuous cellular morphology of finely distributed eutectic Si particles characteristic of PBF-LB/M samples possesses the highest corrosion resistance. Conversely, the breakdown of the Si network for the sample heat-treated at 300 °C results in continuous corrosion along the broken Si network [64]. These findings highlight the critical role of the microstructural scale and morphologies of the Si particles in governing corrosion behaviour.

Consequently, these thermally induced microstructural changes in the AB substrate offset the detrimental effects of roughness and tensile residual stresses in the ESD coating, leading to a convergence in corrosion performance between the two conditions.

## 4. Conclusions

In this work, the combined application of Electrospark Deposition (ESD) and Ultrasonic Peening Treatment (UPT) was investigated as a post-processing strategy for refurbishing Powder Bed Fusion–Laser Beam/Metal (PBF-LB/M)-produced AlSi10Mg components.

The main findings can be summarized as follows:

- The microstructure of the ESD coating was very fine due to the extremely high cooling rate and consists mainly of a cellular structure aligned parallel to the build-up direction. The interfacial adhesion was excellent, with no evidence of discontinuities, voids, or delamination along the bonding line.
- After UPT, porosity decreased by approximately 50%. Analysis of pore size distribution revealed a marked reduction in void density across all size ranges.
- Surface roughness was improved by UPT, leading to a decrease of 78% with respect to the average roughness value of the untreated coating.
- With respect to the as-deposited ESD coating, after UPT, the hardness increased by 48% at the upper edge, 32% in the central region, and 23% near the interface due to UPT.
- Residual stresses in the ESD coating, originally tensile at 121.9 ± 9.5 MPa (0°), 78.5 ± 16.9 MPa (45°), and 78 ± 15.6 MPa (90°), were reversed to compressive values of −59 ± 25.3 MPa, −68.2 ± 30.9 MPa, and −90.8 ± 9.8 MPa, respectively, after UPT.
- The electrochemical investigation demonstrates that PBF-LB/M samples heat-treated at 300 °C and the as-deposited ESD substrate exhibit comparable corrosion behaviour. However, the combined treatment ESD + UPT significantly modifies the electrochemical response of the AlSi10Mg alloy. While the Ultrasonic Peening Treatment induces a shift toward a nobler corrosion potential, it also results in a slightly higher corrosion current density compared to the ESD substrate.

The high quality of the analyzed ESD and UPT deposits demonstrates their suitability for repairing damaged PBF-LB/M components. Consequently, future developments will focus on evaluating the tensile and fatigue properties, alongside detailed pre- and post-corrosion analyses by SEM/EDS, to fully characterize the degradation mechanisms of ESD- and UPT-repaired samples using the optimized process parameters identified in this study.

## Figures and Tables

**Table 1:** AlSi10Mg powder composition (wt.%) [22].

Si	Mg	Fe	Mn	Ti	Cu	Zn	Pb	Sn	Ni	Al
10.20	0.34	0.17	0.01	0.34	0.01	0.01	<0.01	<0.01	<0.01	bal.

**Table 2:** Process parameters for PBF-LB/M.

Power [W]	Exposure Time [µs]	TOFF [μs]	Point Distance [µm]	Hatch Distance [µm]	Layer Thickness [µm]	VED [J/mm^3^]	v [mm/s]	Linear Energy [J/mm]
375	50	20	140	90	30	69	2000	0.19

**Table 3:** Process parameters for Electrospark Deposition.

Voltage [V]	Capacitance [μF]	Frequency [Hz]	Spark Pulse Energy [J]	Power [W]	Electrode Rotation Speed [rpm]
100	150	90	0.75	67.5	1200

**Table 4:** Process parameters for Ultrasonic Peening Treatment.

Energy Level	Application Time [s]	Tested Area [mm^2^]	Working Head
2 (Intermediate)	30	36	Three-pin

**Table 5:** Average surface roughness (Ra) of the investigated AlSi10Mg samples: comparison between as-deposited (ESD) and post-treated (ESD + UPT) conditions.

Roughness	ESD	ESD + UPT
Ra [µm]	21.68 ± 0.41	4.69 ± 0.04

**Table 6:** Deposition void percentage in various circularity ranges before and after UPT.

Circularity Range	Deposition Void Percentage	
	ESD [%]	ESD + UPT [%]
0–0.4	8.3	9.6
0.4–0.8	28.9	37.8
0.8–1	63.0	52.6

**Table 7:** Comparison of results of the potentiodynamic tests (E_corr_ and j_corr_) for the AlSi10Mg samples in the as-built state and subjected to heat treatment at 300 °C for 2h (AB sample), ESD, and ESD + UPT.

Sample	E_corr_ [V vs. Vref]	j_corr_ [A/cm^2^]
AB	−0.74	2.718 × 10^−6^
ESD	−0.74	2.253 × 10^−6^
ESD + UPT	−0.69	1.686 × 10^−5^

## Data Availability

The original contributions presented in this study are included in the article. Further inquiries can be directed to the corresponding author.
